# Significance of Circulating Tumor Cells in Soft Tissue Sarcoma

**DOI:** 10.1155/2015/697395

**Published:** 2015-06-18

**Authors:** Chiara Nicolazzo, Angela Gradilone

**Affiliations:** Dipartimento Medicina Molecolare, Sapienza Università di Roma, Viale Regina Elena 324, 00161 Roma, Italy

## Abstract

Circulating tumor cells can be detected from the peripheral blood of cancer patients. Their prognostic value has been established in the last 10 years for metastatic colorectal, breast, and prostate cancer. On the contrary their presence in patients affected by sarcomas has been poorly investigated. The discovery of EpCAM mRNA expression in different sarcoma cell lines and in a small cohort of metastatic sarcoma patients supports further investigations on these rare tumors to deepen the importance of CTC isolation. Although it is not clear whether EpCAM expression might be originally present on tumor sarcoma cells or acquired during the mesenchymal-epithelial transition, the discovery of EpCAM on circulating sarcoma cells opens a new scenario in CTC detection in patients affected by a rare mesenchymal tumor.

## 1. Introduction

Sarcomas are relatively rare but particularly lethal tumors, with over 5,800 deaths per year in the United States [1], accounting for about 1% of all adult cancers and approximately 20% of pediatric solid malignancies [2]. The word sarcoma represents over fifty subtypes of soft tissue malignant tumors of mesenchymal origin. In 2013, the* World Health Organization (WHO) Classification of Tumors of Soft Tissue and Bone* has been published, according to the pathogenesis of soft tissue and bone tumors related to the histologic and genetic findings [3, 4].

The diagnosis is performed by hematoxylin and eosin staining of sections and immunohistochemistry. To date, radiographic imaging and PET are performed to detect recurrence and metastasis.

However, different technologies are now available for molecular characterization of such tumor types, as recently discussed by Smith et al.; analyses as Polymerase Chain Reaction (PCR), fluorescent in situ hybridization (FISH), Real-Time PCR, and next-generation sequencing (NGS) or the presence of reciprocal chromosomal translocations and fusion genes may be useful for diagnosis and treatment of sarcoma patients [5].

Since most soft tissue sarcomas use the hematogenous dissemination to metastasize to lungs, liver, bones, and subcutaneous tissue whereas a small percentage may spread to lymph nodes [6, 7], it is possible to isolate and characterize circulating tumor cells (CTCs) from whole blood based on their biological and/or physical properties [8].

Furthermore, CTCs provide through their molecular evaluation an example of “liquid biopsy” useful for cancer patient care [9].

## 2. CTC

The definition of CTC is often associated with tumors of epithelial origin, even if this definition may include tumor cells derived from all solid tumors with both epithelial and mesenchymal origin. CTCs can be detected from the peripheral blood of cancer patients; ten years ago the prognostic significance of CTC isolated by CellSearch System among patients with metastatic breast [10–13], colorectal [14, 15], and prostate cancer [16, 17] has been demonstrated.

Circulating tumor cells referred to carcinomas were first described in 1869 by Ashworth [18] at autopsy examination, whereas Engell [19] described for the first time in 1955 the presence of cancer cells in the bloodstream. Every day a primary tumor releases an amount of CTCs of 10^6^ for gr of tumor; the majority of them die and only a small percentage, about 0.01%, survive, thus contributing to metastatic spread [20].

To date, the knowledge derived from years of studies focused on the role of CTCs comes from the analysis of epithelial tumors.

The physical and biological characteristics of CTCs have been employed for their enrichment and isolation; in particular, their larger size compared to blood cells and the presence, on their surface, of epithelial antigens allowed new assays for their isolation and characterization.

## 3. Carcinoma

The discovery of epithelial cell adhesion molecule (EpCAM) expression on epithelial cancer cells opened a new era in the development of several technologies for CTC enrichment and isolation. To date, due to the presence of new sensitive assays we can isolate at least single tumor cells between billions of peripheral blood cells. CellSearch is an EpCAM-based method, the only one approved by the Food and Drug Administration (FDA) for CTC enumeration. CellSearch assay was analytically validated by Allard et al. [21], and it is recognized as a very sensitive, accurate, and reproducible method [21]. The CellSearch kits used for epithelial CTC capture contain ferrofluids labeled with EpCAM, the staining reagents, 4′,6-diamidino-2-phenylindole, dihydrochloride (DAPI), CD45-Allophycocyan (CD45-APC), and cytokeratin 8, 18, and 19 Phycoerythrin (CK-PE) [22]. According to CellSearch manufacturer's instructions, a CTC is characterized by positivity for EpCAM, cytokeratins (CKs), nuclear dye (DAPI), and negativity for CD45 [23]. CTC enumeration, detected in 7.5 mL of blood, has prognostic value in metastatic breast [10–13], prostate [16, 17], and colon cancer patients [14, 15]. For metastatic patients with breast and prostate cancer with a count of CTC fewer than five in a blood sample of 7.5 mL higher survival was observed, as well as for metastatic colorectal cancer patients with a CTC value of 3 or fewer for 7.5 mL of blood. The presence and prognostic significance of CTC in blood of metastatic patients with small and non-small cell lung cancer [24, 25], stomach cancer [26], pancreatic cancer [27], ovarian cancer [28], and both advanced [29] and nonmuscle invasive bladder cancer [30, 31] have been also demonstrated.

In order to isolate CTC from cancer patients affected by carcinoma, studies have advanced from Reverse Transcriptase-Polymerase Chain Reaction- (RT-PCR-) based methods to immunomagnetic enrichment of tumor cells exploiting the presence of molecular markers as EpCAM on the CTC surface.

Immunomagnetic separation (IMS) may be achieved using Dynabeads, through enrichment or depletion of leukocytes with magnetic beads coated anti-CD45 antibody [32, 33]. For the enrichment beads coated with antibodies against specific antigens present on the cell surface, especially towards the human EpCAM or antibody against tumor specific markers can be used.

Among the molecular-based techniques developed, AdnaTest is a series of commercially available assays that combines an immunomagnetic enrichment of tumor cells followed by subsequent multiplex RT-PCR [34].

To date, microfluidic devices for CTC capture called CTC-Chip are also available; in recent years several devices have been created, both EpCAM and non-EpCAM-based, all able to provide living CTCs, suitable for molecular analysis and characterization [35–37].

A simple and versatile assay for CTC capture and genomic or proteomic analysis is ISET. Isolation by size of tumor cells (ISET) is a marker-independent assay for the isolation of intact CTCs from blood through direct filtration, using polycarbonate membrane with 8 *μ*m diameter cylindrical pores taking advantage of the larger size of tumor cells compared with leukocytes. CTCs are then identified by cytomorphology and can be characterized by immunofluorescence (IF) assays but their enumeration is hard to be obtained [38, 39].

## 4. EMT

The epithelial-mesenchymal transition (EMT) has been widely investigated in carcinomas. Being characterized by loss of epithelial markers and acquisition of mesenchymal features, it allows carcinoma cells to escape from their structural confines, increasing their mobility and invasiveness, to enter into the bloodstream and to adhere and develop into distant metastases [40, 41]. Cells that undergo EMT are different from the conventional concept of CTCs: they express low or null EpCAM expression and might be missed by EpCAM-based approach [42–44]. Progression of solid tumors involves spatial and temporal occurrences of EMT, whereby tumor cells acquire a more invasive and metastatic phenotype. Subsequently, the disseminated mesenchymal tumor cells undergo the mesenchymal-to-epithelial transition (MET), at the site of metastases, which often recapitulate the pathology of their corresponding primary tumors. EMT is considered a reversible process of switch from an epithelial to a mesenchymal cellular phenotype. This transition leads to downregulation of epithelial markers as CKs and E-cadherin and to upregulation of mesenchymal biomarkers as vimentin, fibronectin, and N-cadherin [40, 45, 46]. EMT enhances migratory and invasive properties of cancer cells and their survival in the bloodstream. The following MET process is responsible for CTC extravasation and colonization in distant organs lesion which is expressed with metastatic disease [47–49].

## 5. Carcinosarcoma

Carcinosarcomas are rare biphenotypic tumors with characteristics of epithelial as well as sarcoma tumor cells [50, 51]. The hypothesis that stem/progenitor cells can play a role as common precursors for tumors of carcino/sarcoma phenotype such as squamomelanocytic tumors [52] and perhaps carcinosarcomas has been suggested.

Carcinosarcoma was originally described in 1972 by Dawson [53] and more recently studies on lung, ovarian, and uterine carcinosarcomas were performed. Paniz Mondolfi et al. [51] recently published a study performed on a primary cutaneous carcinosarcoma and demonstrated an immunoreactivity for EpCAM in both mesenchymal and epithelial components, suggesting that EpCAM might represent a potential target for cutaneous carcinosarcoma.

The presence, in carcinosarcoma tumors, of sarcomatous and epithelial tumor components led to further studies focused on the presence of epithelial markers on sarcoma cells.

## 6. Sarcoma

Few studies have been performed on sarcoma-derived CTCs, perhaps because sarcomas are considered relatively rare neoplasms despite their poor prognosis.

More recently, EpCAM-positive circulating cells were detected in soft tissue sarcoma patients and a genomic meta-analysis of gene expression profiles demonstrated that EpCAM mRNA is expressed in different sarcoma cell lines. Immunohistochemical staining revealed EpCAM protein expression in a subset of angiosarcomas and leiomyosarcomas and in all the osteosarcomas analyzed; in addition, a negative prognostic marker for leiomyosarcoma patients has been suggested [54].

The presence of EpCAM on CTC from metastatic sarcoma patients was shown by Vincenzi et al. using the CellSearch System. They enrolled twenty-three patients with metastatic STSs at first, second, and third line of chemotherapy and CTCs were detected in 43% of enrolled patients with a CTC count range of 0–4 cells/7.5 mL [8].

In a recent review by Chang et al. [55], CTC detection in sarcoma has been underlined; the majority of studies were performed on Ewing's sarcoma by RT-PCR analysis for the research of the fusion gene product associated with the disease EWS-FLI-1 and EWS-ERG marker. Data regarding the prognostic significance of CTC isolation in sarcoma, although poor, show a correlation between CTC presence and poor outcome or disease progression [56].

Cancer cells show a larger size compared to leukocytes and this physical characteristic was used to isolate and characterize CTC from sarcoma tumors for the first time by Chinen et al. [56]. In this study, which was performed on eleven patients with metastatic/recurrent or locally advanced soft tissue sarcomas, positivity for CTC detection has been shown for all patients enrolled, with a recovery rate of CTC from two to 48 per 8 mL of blood. For CTC characterization authors used anti-CD45 (specific marker for WBCs), anti-vimentin (mesenchymal-related marker), and anti-Pan CK (epithelial-related marker) antibodies, to discriminate CTCs from leucocytes in the immunocytochemistry assay.

Vimentin is considered the main protein associated with the EMT process and consequently with increased invasiveness, chemoresistance, and metastatic phenotype of these cells. The presence of vimentin on the surface of cancer cells is considered a sign of a mesenchymal phenotype of CTCs [57].

Although detection of cell-surface vimentin in cancer cells has been demonstrated in different studies [58, 59], its role as a biomarker by using a monoclonal antibody 84-1 for detecting CTC from blood of cancer patients affected by carcinoma as well as sarcoma has been established [57].

## 7. MET

If it is true that EMT has been suggested to promote the growth of epithelial tumor cells at distant sites during metastasis, the inverse process MET might be considered as an efficient process for the dissemination of sarcoma cells.

Several types of sarcomas, including synovial sarcoma, leiomyosarcoma, chondrosarcoma, osteosarcoma, chordoma, epithelioid sarcoma, and ES/PNET, have been reported to show epithelial differentiation on the basis of detection of epithelial markers including E-cadherin. Recent findings reveal that mesenchymal-epithelial transition may exist in sarcomas, leading to a switch towards an epithelial phenotype. Accumulating evidences suggest that deeper investigation and understanding of MET in sarcomas would shed light on the pathogenesis of sarcomas and might lead to identification of potential clinical biomarkers for prognosis and targets for sarcoma therapeutics.

In this view, EMT/MET processes may be considered the link between carcinoma and sarcoma tumors, two distinct and reversible regulated mechanisms employed by tumoral cells, both responsible for developing distant metastases.

## 8. Discussion and Conclusion

Although it is not clear whether tumor sarcoma cells constitutively express EpCAM or alternatively they acquire it during the mesenchymal-epithelial transition, the discovery of EpCAM on circulating sarcoma cells opens a new scenario in CTC detection in patients affected by rare tumors.

CellSearch System offers the possibility to study the expression of an additional marker of interest by the use of the fourth channel. To date, this channel has been used to study the apoptotic status of CTCs integrating the system with a monoclonal antibody, anti-M30 [60], anti Bcl-2 [61], and anti-M65 [62].

CellSearch, an EpCAM-based assay, can be integrated with an anti-vimentin antibody in its fourth channel, to discriminate sarcoma circulating cells positive for EpCAM and vimentin and negative for CD45, from leucocytes positive for CD45.

Vimentin is considered a specific marker of mesenchymal derivation or differentiation and its expression in sarcoma circulating cells, presumably undergoing MET, seems contradictory and requires further studies.

Satelli et al. [63] developed an anti-vimentin antibody able to discriminate the expression of cell-surface vimentin, mainly associated with cancer cells, from the intracellular vimentin expressed by most white blood cells in order to exclude bias to use this protein as a CTC marker.

Using the CellSearch System this bias could be excluded: the choice of intracellular or extracellular antibody is indifferent because we may discriminate the false positive results by the leucocytes positivity for CD45.

More than ten years ago, CTC enumeration was validated as prognostic biomarker in metastatic breast cancer using the US Food and Drug Administration- (FDA-) cleared system CellSearch (Janssen Diagnostics, LLC, Raritan, NJ, USA); we believe that studies on biomarkers in sarcoma patients may quickly evolve in the next future through CTC enumeration as well as phenotypic and genotypic characterization.
